# Spectrum Effect Relationship and Component Knock-Out in *Angelica Dahurica* Radix by High Performance Liquid Chromatography-Q Exactive Hybrid Quadrupole-Orbitrap Mass Spectrometer

**DOI:** 10.3390/molecules22071231

**Published:** 2017-07-21

**Authors:** Jinmei Wang, Linna Peng, Mengjun Shi, Changqin Li, Yan Zhang, Wenyi Kang

**Affiliations:** 1 Institute of Chinese Materia Medica, Henan University, Kaifeng 475004, China; wangjinmeiscp@126.com (J.W.); penglinna1991@163.com (L.P.); 18237879687@163.com (M.S.); xiaoya0204@163.com (C.L.); 2 Kaifeng Key Laboratory of Functional Components in Health Food, Henan University, Kaifeng 475004, China; 3 Hebei Food Inspection and Research Institute, Shijiazhuang 050091, China; snowwinglv@126.com

**Keywords:** *Angelicadahuricae*, tyrosinase, spectrum-effect relationship, component knock-out

## Abstract

Different extracts of *Angelica dahuricae* were available for whitening or treating vitiligo clinically. They showed inhibitory or activating effects on tyrosinase, a rate-limiting enzyme of melanogenesis. This study aimed to identify active compounds on tyrosinase in water extract of *Angelica dahurica* Radix. We applied spectrum-effect relationship and component knock-out methods to make it clear. HPLC was used to obtain the specific chromatograms. The effects on tyrosinase activity were examined by measuring the oxidation rate of levodopa in vitro. Partial least squares method was used to examine the spectrum-effect relationships. The knocked-out samples were prepared by HPLC method, and the identification of knocked-out compounds was conducted by the high performance liquid chromatography-four stage rod-electrostatic field orbit trap high resolution mass spectrometry. Results showed that S6, S14, S18, S21, S35, S36, S37, S40, and S41 were positively correlated to inhibitory activity of *Angelica dahuricae* on tyrosinase whereas S9, S11, S8, S12, S22, and S30 were negatively correlated. When the concentration of each sample was 1 g·mL^−1^, equal to the amount of raw medicinal herbs, oxypeucedanin hydrate, imperatorin, cnidilin, and isoimperatorin had inhibitory effects on tyrosinase activity whereas byakangelicin and bergapten had activating effects.

## 1. Introduction

As a common ingredient in the Asia traditional medicine, *Angelica dahurica* Radix (AD) is the dried root of *Angelica dahurica* (Fisch. ex Hoffm.) Benth. et Hook. f. or *A. dahurica* (Fisch. ex Hoffm.) Benth. et Hook. f. var. Formvsana (Boiss) Shan et Yuan. Especially in China, AD is one of the common traditional Chinese medicines (TCM), which has been used for the treatment of wind-cold type of common cold, headache, rhinitis, and toothache and has been officially listed in the Chinese Pharmacopoeia [1].

Coumarin is the main chemical components in AD [2,3,4]. The reported studies have shown that a majority of coumarins in AD are photosensitive and can be used for the treatment of hypopigmentation disease because of their photosensitization [5,6]. For instance, the compound Angelica Tincture, which is widely used in the treatment of vitiligo clinically, is prepared by taking AD and Psoraleae Fructus as the principal ingredients and then dipping them in 70% ethanol after being powdered [7].

Tyrosinase is the key rate-limiting enzyme in melanin biosynthesis pathway [8,9]. Interestingly, photosensitive furocoumarin extracted from AD can enhance tyrosinase activity, thus increasing the synthesis of melanin and achieving the treatment of vitiligo [7]. However, clinical practice and research have shown that AD also has whitening effect. According to the traditional Chinese medical science theory, the whitening TCMs can be used to treat skin disease caused by hyperpigmentation. AD is one of the whitening TCMs and is selected to make up the famous classical prescription for whitening named “Seven-White Ointment”, which was historically recorded as “Tai Ping Sheng Hui Fang” in Song Dynasty, “Yu Yao Yuan Fang” in Yuan Dynasty, and medicine “Pu Ji Fang” in Ming Dynasty). All of the water extracts of the TCMs in this prescription (except the egg white) had the inhibitory effect on tyrosinase activity [10].

The bidirectional regulation of AD on melanogenesis is closely related to the extract fractions and concentration [11,12,13,14,15]. There may be the components in AD with inhibitory and activating effects on tyrosinase activity, respectively. In order to prepare the components with different influences on tyrosinase activity in AD clear, the method of spectrum-effect relationship which is considered to be a systematic approach to TCM research was adopted firstly [16]. We conducted study on the spectrum-effect relationship of TCMs by establishing mathematical model to connect the characteristic peaks with the pharmacodynamic value, and to explore the correlation between them, so as to provide a reliable method for elucidating the material basis of TCMs. In this study, we prepared the sample by water decoction method, which is commonly used for TCM clinically, and then established the chromatographic fingerprint of AD and the evaluation model of effect on tyrosinase activity in vitro, analyzed the relationship between the characteristic peaks with the pharmacodynamic value with the established mathematical model, fitted the active components related to inhibition on tyrosinase, so as to provide reference for the pharmacodynamic material basis of AD. Secondly, we applied component knock-out method to find out active components of AD on tyrosinase activity for a more comprehensive research. Pharmacodynamic material basis identification model of TCM based on component knock-out suggest that the target component will be knocked out from the complete sample and the changes in the efficacy of the samples before and after the target component is knocked-out can reflect the contribution of the target component to the efficacy of the TCM. By this way, the interactions between the target component and other components can also be investigated. The continued study was guided by the result of spectrum effect relationship research. High performance liquid chromatography (HPLC) method was used to “knock out” and obtain the target components and negative samples. The effects of target components, negative samples and water extracts of AD on tyrosinase activity were obtained by the evaluation model of effect on tyrosinase in vitro and compared in parallel, so as to identify the components in AD related to the activity and their interactive effects on tyrosinase activity.

Recent studies showed that a significant portion of small-molecule drugs act on enzymes. Because enzymes are such important drug targets, it is not a surprise that constantly updated technologies and ideas have been performed with enzymes. High throughput screening is one of the most frequently used methods of enzyme analysis that can be defined as the implementation of assays in the wells of microplates in combination with liquid handling robotics [17]. Our study drew on the idea of high throughput screening but made with adjustments. It was performed with the spectrum effect relationship research reoriented and the component knock-out method for verification, to achieve the high-throughput enzyme analysis of different compounds in AD with more purposiveness.

## 2. Results and Discussion

### 2.1. Spectrum-Effect Relationships

#### 2.1.1. Determination of Quantitative Chromatographic Peaks

The previous “spectrum-effect relationship” researches were based on different fields, origins, harvest time, processing method and batches of TCM etc. [18,19,20,21,22,23]. However, there have been great changes that have taken place after a long-term regional difference on TCM. Then the types and contents of trace elements and chemical components have also been changed. These changes may lead to subtle changes in some pieces of information but may play a decisive role miss after common peaks matched from HPLC specific chromatograms [24]. Our previous studies showed that the active components were changed in the TCMs processed through the classical constant temperature method. Therefore, this study used the classical constant temperature method to carry on the processing with different methods to the same batch of AD, to eliminate the disturbance from the origin, the batch and other factors, and to screen out more accurately the material basis of AD on tyrosinase activity.

**Figure 1:**
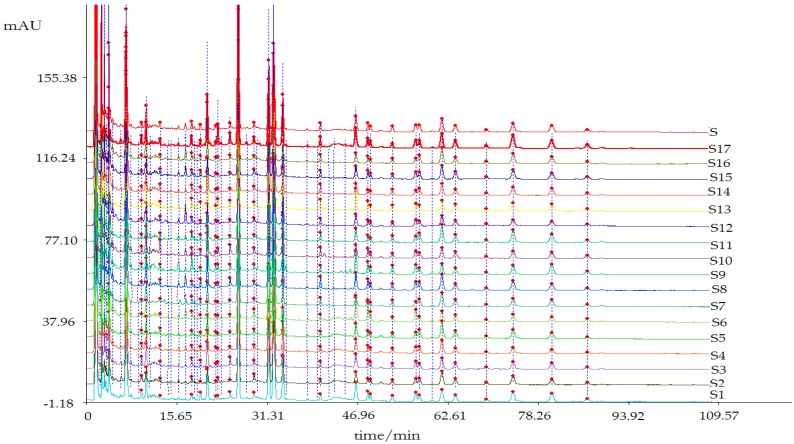
The matching HPLC characteristic chromatograms of different treatments of AD.

The quantitative chromatographic peaks were determined by the software < Chinese traditional medicine chromatographic fingerprint similarity evaluation system 2004, 1.0 A Edition > Multi-point correction of chromatographic peak position was performed based on chromatographic peaks, which were found in each sample with good separation by reference to the chromatogram of crude AD water extract. A contrast chromatogram was generated by average method. The matching chromatograms and peak areas were shown in Figure 1 and Table 1, Table 2, Table 3 and Table 4.

#### 2.1.2. Determination of Inhibitory Effects of AD Water Extract on Tyrosinase in Vitro

Effect of each sample on tyrosinase activity is shown in Table 5. When the sample concentration was 1 g·mL^−1^, equal to the amount of raw medicinal herbs, each sample showed inhibitory effect on tyrosinase activity, and inhibitory effect on the tyrosinase activity of the samples prepared from heated AD were significantly lower than that of the crude AD (*p* ≤ 0.001). With the decrease in sample concentration, samples of crude AD, 50 °C-54 h, 50 °C-162 h, 60 °C-18 h, and 60 °C-54 h showed inhibitory effects, which was decreased at first and then increased. Samples of 50 °C-216 h, 60 °C-72 h, 70 °C-6 h, 70 °C-24 h, 80 °C-4 h, and 80 °C-6 h exhibited inhibitory effects at the highest concentration, but showed activation in the intermediate concentration, and showed inhibitory effect at low concentration. Samples of 60 °C-36 h, 70 °C-12 h, 70 °C-18 h and 80 °C-2 h showed inhibition in the high concentration, but became activation when the concentration was decreased. It can be inferred according to this result that different heating conditions had different effects on components in AD, which had either inhibition or activation effect on tyrosinase activity, and thus led the different samples to show different effects on tyrosinase activity. Moreover, the effect of compounds in AD on tyrosinase activity displayed various dose-response relationship, when the sample concentration was changed, part of sample showed bidirectional regulation on tyrosinase activity.

#### 2.1.3. The Regression Equation of Partial Least Squares Analysis

Partial least squares regression analysis is a multivariate regression model co-inhering of multivariate data fusion and principal component analysis. This kind of analysis method has advantages, such as low computational complexity and high prediction accuracy, without excluding samples, easy to qualitatively explain. Furthermore, it also can maximize the use of limited data with high predictability [25]. In our study, the quantitative chromatographic peaks area was set as the independent variable (X), inhibition rate of tyrosinase activity (at the concentration of 1 g·mL^−1^) as the dependent variable (Y). The regression equation was expressed as follows:

Y = 0.009474 X1 − 0.039034 X2 + 0.045899 X3 − 0.062253 X4 + 0.070846 X5 + 0.144735 X6 - 0.038385 X7 − 0.186504 X8 − 0.216749 X9 + 0.009362 X10 − 0.133367 X11 − 0.109319 X12 - 0.007779 X13 + 0.142509 X14 + 0.051088 X15 + 0.019396 X16 + 0.053583 X17 + 0.101717 X18 + 0.046538 X19 − 0.003983 X20 + 0.335489 X21 − 0.358561 X22 + 0.060104 X23 − 0.077885 X24 − 0.080212 X25 − 0.084729 X26 − 0.031941 X27 − 0.022003 X28 + 0.066404 X29 − 0.124915 X30 + 0.011693 X31 + 0.016945 X32 + 0.003708 X33 + 0.032733 X34 + 0.107463 X35 + 0.249803 X36 + 0.139539 X37 − 0.088576 X38 + 0.004961 X39 + 0.120817 X40 + 0.120802 X41 − 0.036626 X42(1)

**Figure 2:**
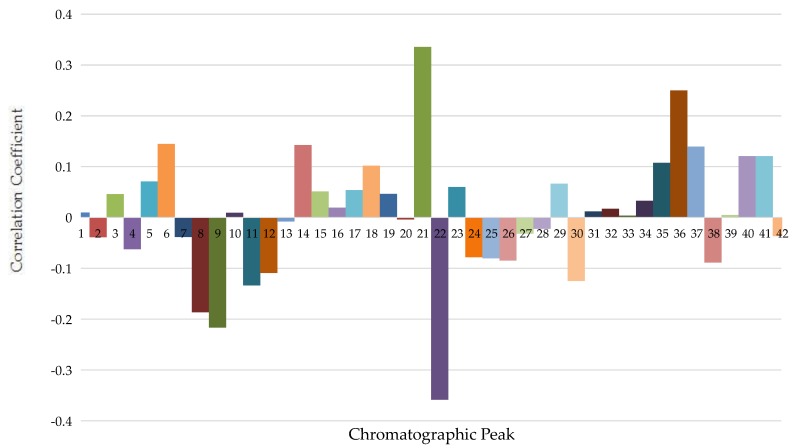
Standardization regression coefficient of PLSR equations of AD.

The regression coefficients of partial least squares regression equation were shown in Figure 2. Chromatographic peaks S6, S14, S18, S21, S35, S36, S37, S40, and S41 were positively correlated to inhibitory effects on tyrosinase activity and the correlation coefficient is higher, meaning that when the contents of compounds are increased, the areas of these representative peaks are increased, the inhibitory effects of samples on tyrosinase ability will become stronger. Chromatographic peaks S8, S9, S11, S12, S22, and S30 were negatively correlated to inhibitory effects on tyrosinase activity, and the absolute value of correlation coefficient were higher. Meanwhile, when the content of these compounds were increased, the areas of representative peaks were increased, inhibition capability of the samples on tyrosinase activity would be weaker.

### 2.2. Component Knock-Out Methods

#### 2.2.1. HPLC Chromatogram of Knock-Out Components and Negative Samples

The chromatogram knock-out method is commonly used for its simple operation [26], rapid preparation and high precision. It is very suitable for polar and weak polar and non-polar compounds based on liquid-liquid partition chromatography. However, its separation effect is limited because of its dependence on device. In order to obtain more precise separation, the analytical HPLC was chosen. We obtained a small amount of target component and negative sample last, owing to the limited quantity of the injected sample.

**Figure 3:**
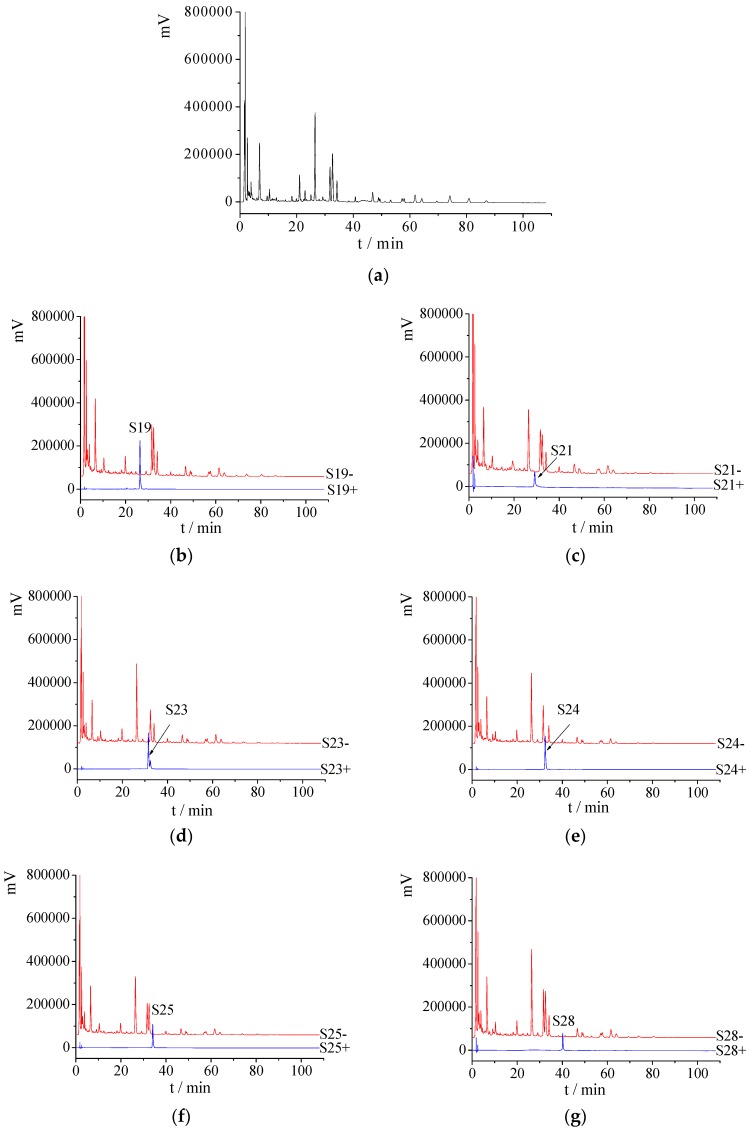

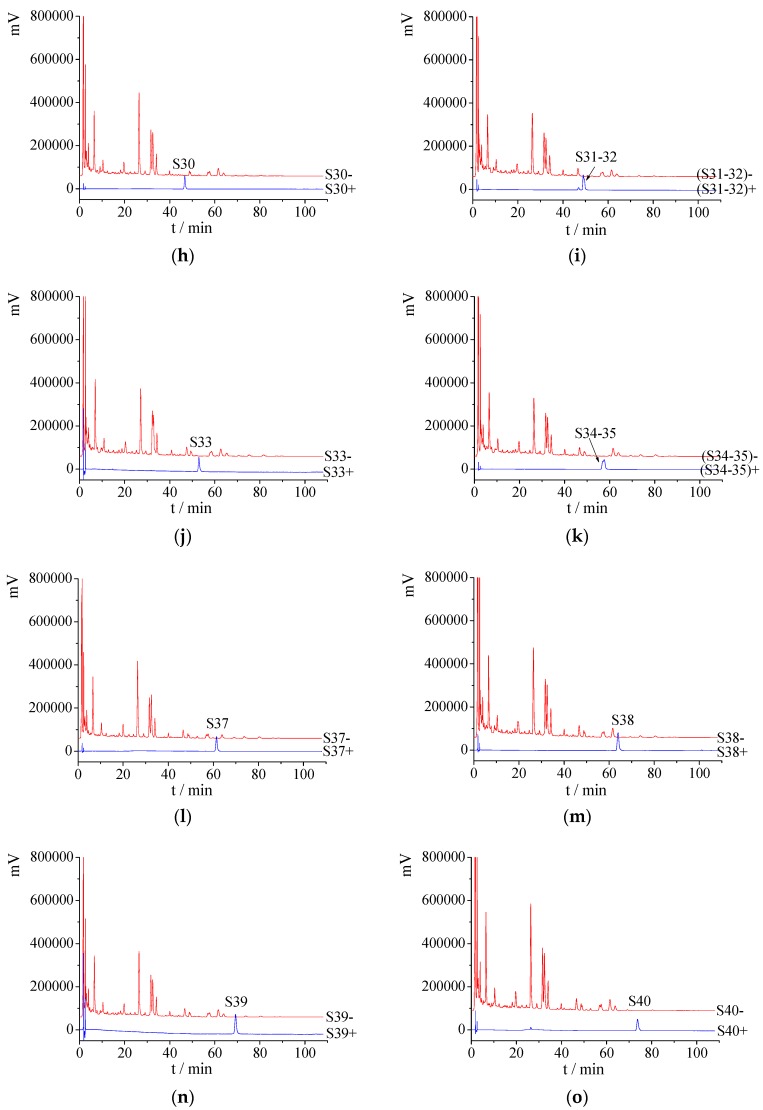

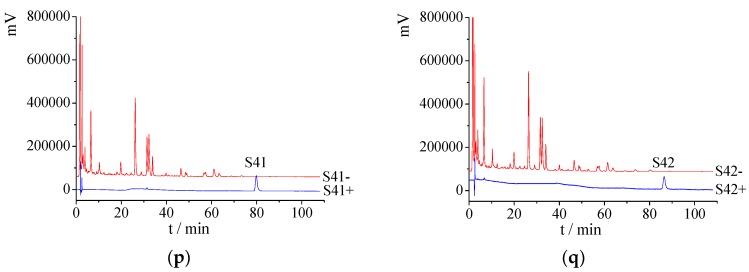
(**a**) HPLC chromatogram of the water extract from AD; (**b**–**q**) HPLC chromatograms of each peak knocked-out component (Sx+) and negative sample (Sx−).

HPLC chromatogram of the water extract of crude AD, knock-out components and negative samples was shown in Figure 3. The purity of these target knocked-out components was high and they could not be found in the negative samples on the whole.

#### 2.2.2. Component Identification

**Figure 4:**
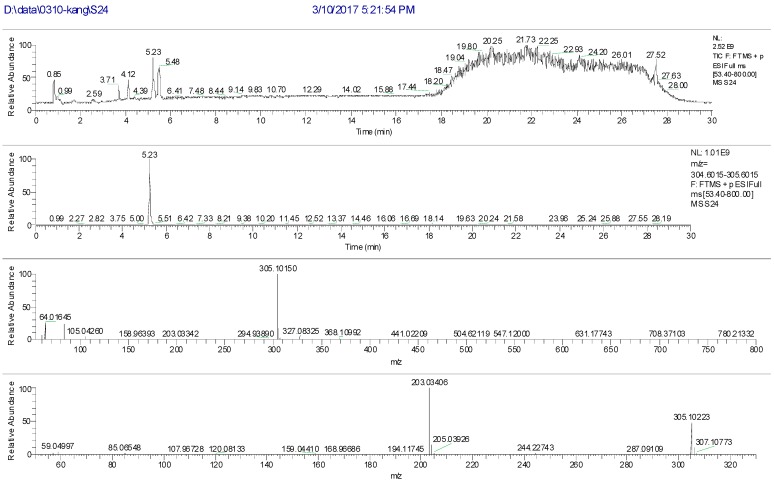
The high resolution mass spectra of Peak 24 knocked-out component.

**Figure 5:**
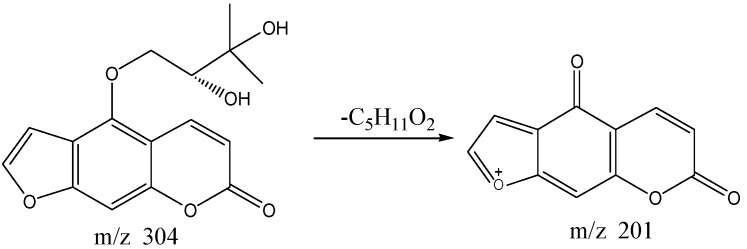
The proposed fragmentation pathway of Oxypeucedanin hydrate.

The high performance liquid chromatography four stage rod-electrostatic field orbit trap high resolution mass spectrometry analysis results of S24 knocked-out component were shown in Figure 4. The retention time was 5.23 min with *m*/*z* 304 and formula C_16_H_16_O_6_. MS/MS spectrum showed that there were ion fragments 304/201. According to the fragmentation pathway (Figure 5), the knocked-out component of S24 was Oxypeucedanin hydrate, presumably.

**Figure 6:**
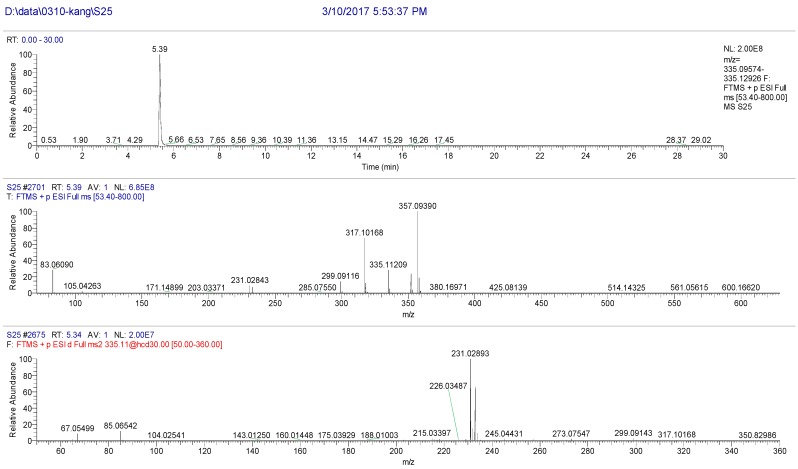
The high resolution mass spectra of Peak 25 knocked-out component.

**Figure 7:**
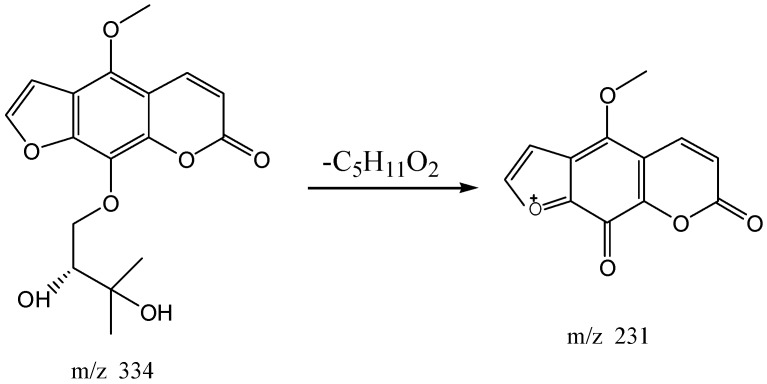
The proposed fragmentation pathway of Byakangelicin.

The high resolution mass spectrum analysis results of S25 knocked-out component were shown in Figure 6 with *m/z* 304 and formula C_17_H_18_O_7_. MS/MS spectrum showed that there were ion fragments with large abundance 231. According to the fragmentation pathway (Figure 7), the knocked-out component of S25 was Byakangelicin.

**Figure 8:**
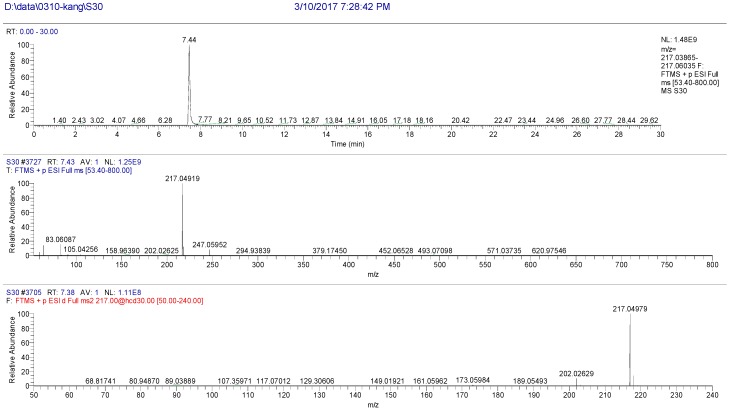
The high resolution mass spectra of Peak 30 knocked-out component.

**Figure 9:**
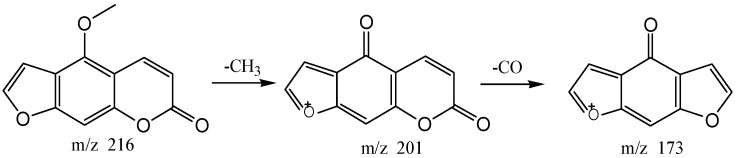
The proposed fragmentation pathway of Bergapten.

The high resolution mass spectrum analysis results of S30 knocked-out component were shown in Figure 8 with *m/z* 216 and formula C_12_H_8_O_4_. MS/MS spectrum showed that there were ion fragments 216/201. According to the fragmentation pathway (Figure 9), the knocked-out component of S25 was Bergapten.

**Figure 10:**
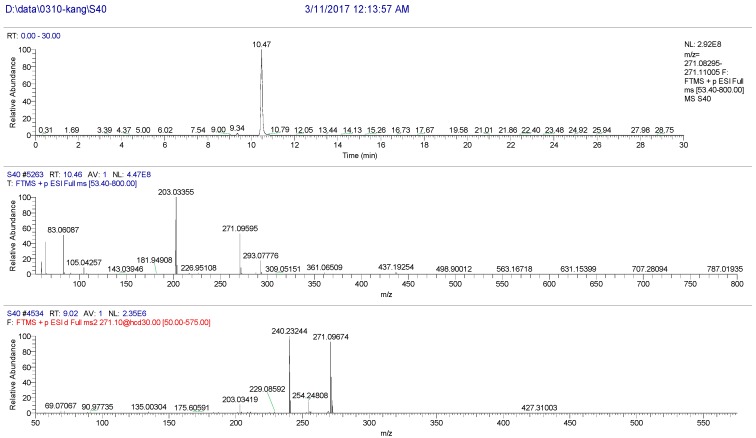
The high resolution mass spectra of Peak 40 knocked-out component.

**Figure 11:**
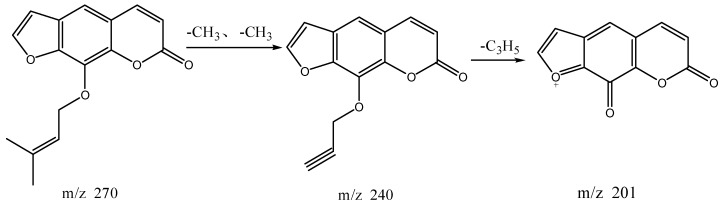
The proposed fragmentation pathway of Imperatorin.

The high resolution mass spectrum analysis results of S40 knocked-out component were shown in Figure 10 with *m/z* 270 and formula C_16_H_14_O_4_; MS/MS spectrum showed that there were ion fragments 270/240/201. According to the fragmentation pathway (Figure 11), the knocked-out component of S25 was Imperatorin.

**Figure 12:**
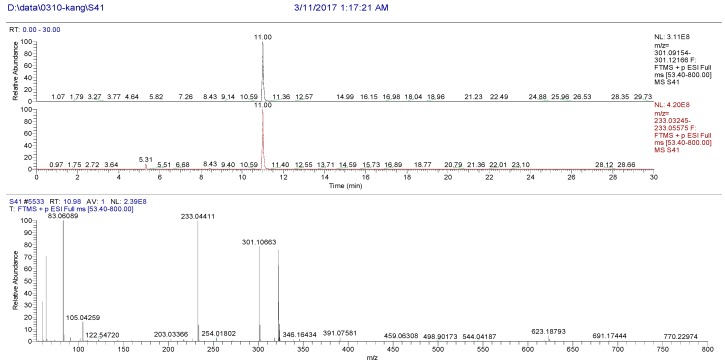
The high resolution mass spectra of Peak 41 knocked-out component.

**Figure 13:**
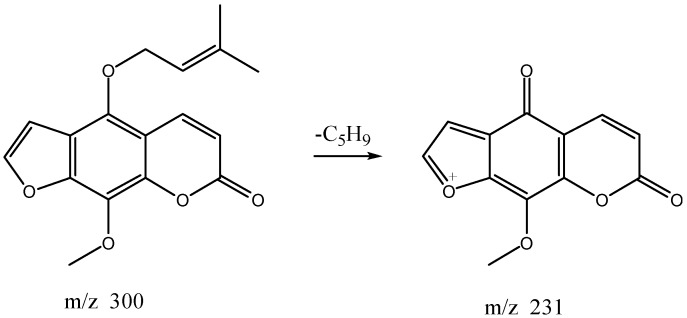
The proposed fragmentation pathway of Cnidilin.

The high resolution mass spectrum analysis results of S41 knocked-out component were shown in Figure 12 with *m/z* 300 and formula C_17_H_16_O_5_; MS/MS spectrum showed that there were ion fragments 300/231. According to the fragmentation pathway (Figure 13), the knocked-out component of S25 was Cnidilin.

**Figure 14:**
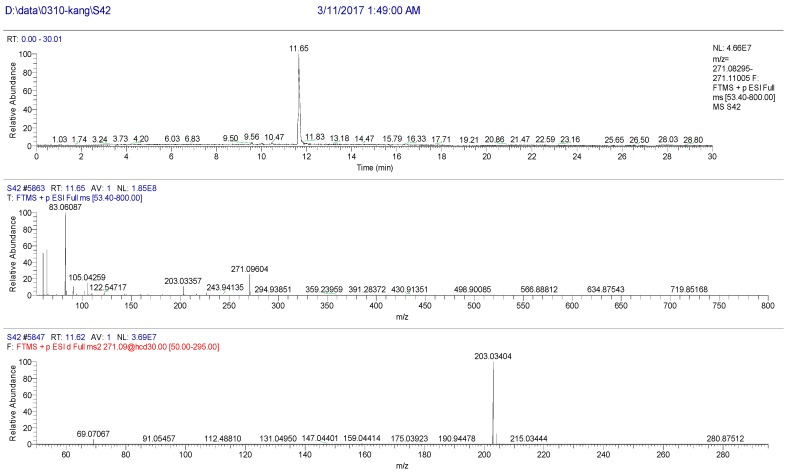
The high resolution mass spectra of Peak 42 knocked-out component.

**Figure 15:**
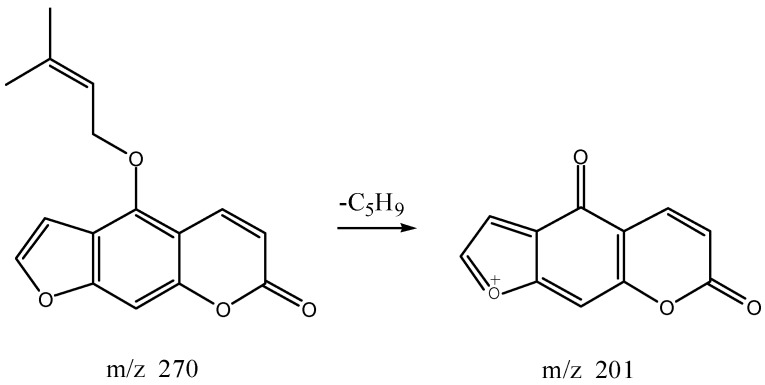
The proposed fragmentation pathway of Isoimperatorin.

The high resolution mass spectrum analysis results of S42 knocked-out component were shown in Figure 14 with *m/z* 270 and formula C_16_H_14_O_4_. MS/MS spectrum showed that there were ion fragments 270/201. According to the fragmentation pathway (Figure 15), the knocked-out component of S25 was Isoimperatorin.

#### 2.2.3. Effect of Knocked-out Components and Negative Samples on Tyrosinase Activity in Vitro

As shown in Table 6, when the concentration of each sample was 1 g·mL^−1^ equal to the amount of raw medicinal herbs, the target components of S23, S24, S28, S34-35, S37, S40, S41 and S42 displayed the inhibitory effect on tyrosinase activity whereas the target components of S19, S21, S25, S30, S31-32, S33, S38 and S39 possessed the activation effect on tyrosinase activity.

**Figure 16:**
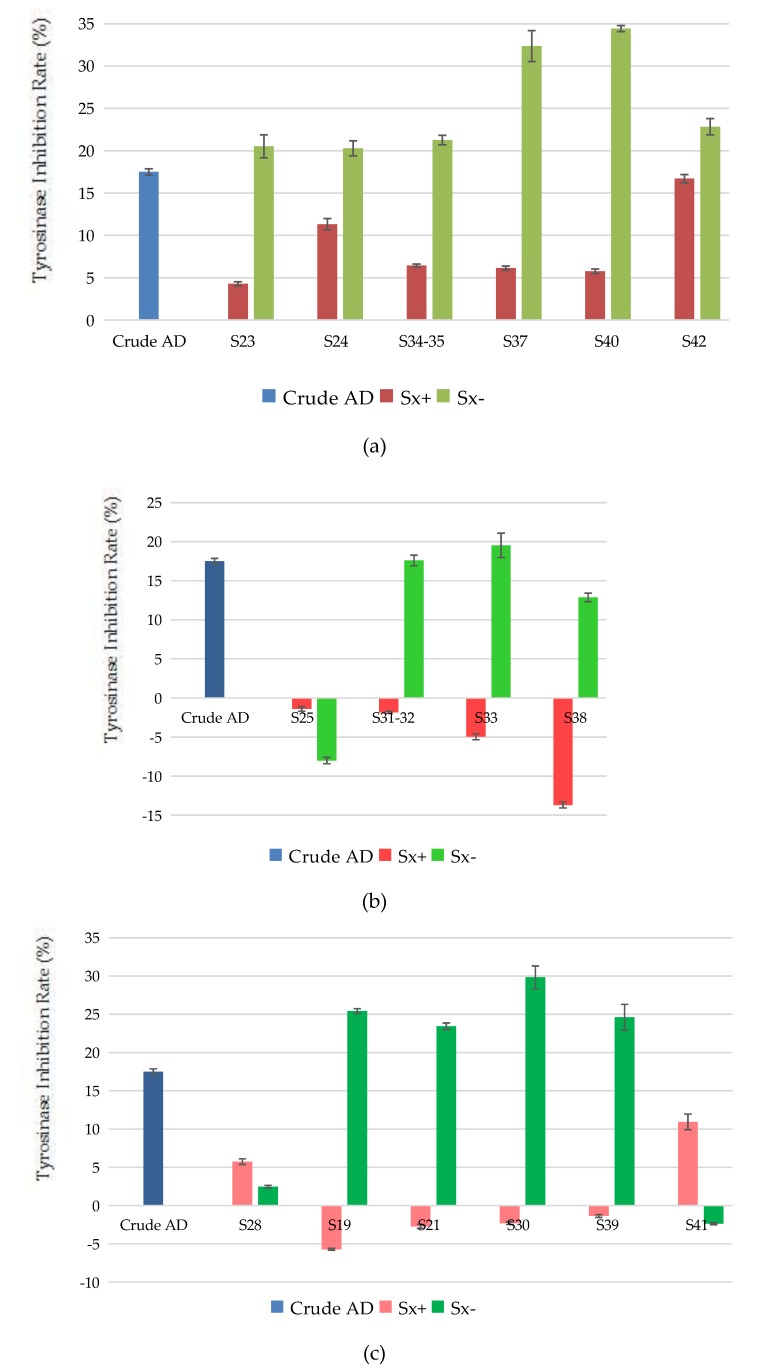
Effect between knocked-out components and negative samples of the water extract of AD: (**a**) Antagonistic effect on tyrosinase inhibition effect; (**b**) Antagonistic effect on tyrosinase activation effect; (**c**) Synergetic effect.

As shown in Figure 16a, the target components and negative samples of S23, S24, S34-35, S37, S40 and S42 both had an inhibitory effect on tyrosinase activity, which were consistent with the effect of crude AD. The sum of the inhibition rate of the target component and negative sample on tyrosinase activity were higher than that of the crude AD, meaning that there may exist antagonistic effect between the target components and components in negative samples on tyrosinase activity. The target components of S24, S40, S42 components were oxypeucedanin hydrate, imperatorin and isoimperatorin, respectively.

As shown in Figure 16b, target components of S31-32, S33, and S38 had activation effect on tyrosinase activity, while the negative samples had inhibitory effect, and sum of inhibition rates of target components and negative samples were lower than the of the crude AD, suggesting that there may exist antagonistic effect on tyrosinase activation effect between the target components and components in negative samples. Both the target component and negative sample of S25 had activation effect on tyrosinase activity, contrary to the crude AD, meaning that there may be strong antagonistic effect on tyrosinase activation effect between the target components and components in negative samples, thus causing the components with inhibitory effects to play the primary role in conferring the effect on tyrosinase activity. Among them, the target component of S25 was byakangelicin. 

As shown in Figure 16c, both the target component and negative sample of S28 possessed inhibitory effect on tyrosinase activity, and the sum of inhibition rates of target components and negative samples were lower than that of the crude AD. It can be inferred that there may be synergetic inhibitory effect between the target components and components in negative samples on tyrosinase activity. The target components of S19, S21, S30 and S39 had activation effect on tyrosinase, but the negative samples had inhibitory effect on tyrosinase and the sum of inhibition rates of target components and negative samples were greater than that of the crude AD, meaning that there may exist synergetic activation effect on tyrosinase activity between the target components and components in negative samples. The target component of S41 had inhibitory effect on tyrosinase activity, but the negative sample showed activation effect on tyrosinase activity. It can be inferred that the target component of S41 makes a greater contribution to the inhibition on tyrosinase activity, and had synergetic inhibitory effect with the components on tyrosinase activity in the negative samples. The target components of S30 and S41 were bergapten and cnidilin, respectively.

Tyrosinase, a vital enzyme, plays an important role in melanin synthesis and neuromelanin formation. Proper content of melanin is important, but excess production of melanin lead to hyperpigmentation. Therefore, tyrosinase inhibitors have been used to prevent hyperpigmentation disorders. Also, tyrosinase is involved in the process to maintain the appearance and nutritional value of many fresh-cut products. Tyrosinase inhibitors are very important in medicine, cosmetics and agriculture. Phenolic compounds, especially polyphenols, flavonols have been revealed to be the strongest inhibitors of tyrosinase [27]. 9-hydroxy-4-methoxypsoralen, a tyrosinase inhibitor, was isolated from AD [28]. In this paper, we identified components in *A. dahurica* that can inhibit or active tyrosinase activity, furthermore, it provided basis for the pertinence of AD to targete to the whitening effect or treatment of vitiligo.

## 3. Materials and Methods

### 3.1. Materials

Acetonitrile was chromatographic grade. Glacial acetic acid was analytical grade. The pure water was purchased from Hangzhou Wahaha Baili Food Co., Ltd., (Hangzhou, Zhejiang, China). (l-3-(3,4-Dihydroxyphenyl) alanine was obtained from Alfa Aesar (Shanghai, China); Tyrosinase was from Worthington Biochemical Corporation (Lakewood, NJ, USA).

### 3.2. Plant Materials

Angelicae dahuricae Radix, identified by Professor Changqin Li of College of Pharmacy, Henan University (Kaifeng, Henan), were purchased in October 2014 in Yuzhou, Henan Province.

### 3.3. Classical Constant Temperature Method

AD was uniformly packed and weighted, then placed in 4 constant temperature drying box. The heating temperature and time were set (Table 7). For each heating time point, 3 parallels were used. When heating process ended, the samples were cooled to room temperature and weighed.

### 3.4. Extraction

Fragments of each sample (about 2 g) was put in a test tube after being weighted precisely, 10 times amount (*w*:*v*) of distilled water was added in it. After being soaked for 30 min, the solution was heated to boiling and kept faint boiling for 30 min by water bath, then 4 layers of gauze was used to remove filter residue. 6 times the amount of distilled water (*w*:*v*) was added into residue and process was repeated. The filtrates from two times filtration were combined, dried, and then prepared to solution, which was equivalent to the amount of raw medicinal herbs at the concentration of 1 g·mL^−1^ by 50% methanol solution.

### 3.5. HPLC Analysis

A LC-20AT HPLC system was obtained from Shimadzu (Kyoto, Japan), and equipped with a degasser, a quaternary gradient low pressure pump, the CTO-20A column oven, a SPD-M20A UV-detector and a SIL-20A automatic sampler. All of the solutions were filtered through the 0.22 μm microporous membrane before they were injected into HPLC system.

Chromatography was performed with an InertSustain RP-C18 column (4.6 mm × 150 mm, 5 μm) at a column temperature of 30 °C The mobile phase was a mixture of acetonitrile (A)-0.1% glacial acetic acid-water (B). The gradient elution steps were set as shown in Table 8 and the flow rate was set at 1.0 mL·min^−1^. The UV detection wavelength was set at 312 nm with the sample volume of 30 μL.

### 3.6. Tyrosinase Inhibition Assay In Vitro

Water extracts of AD were dissolved in 50% methanol solution, and stored at 4 °C in refrigerator. 

Tyrosinase inhibition assay was performed in a 96-well microplate format using Multiskan MK3 microplate reader (Thermo Electron) according to the method reported by Zhang [29]. The compounds were screened for the inhibitory effects on tyrosinase activity using levodopa (LOP) as substrate. 45 μL of K-phosphate buffer (pH 6.8), and 25 μL of mushroom tyrosinase (TYR, 0.2 U·mL^−1^) were incubated with 5 μL of sample at 30 °C for 10 min in water-jacket thermostatic incubator (Sumsung GRP-9270). Then LOP (0.5 mmol·L^−1^) was added to the reaction mixture and incubated at 30 °C for 5 min. The enzymatic reaction was monitored by measuring the change in absorbanceat 492 nm (A_492_) (at 30 °C) due to the formation of the dihydroxyphenylalanine (DOPA) chrome for 5 min. The percentage of inhibition of the enzymatic activity was calculated as follows: tyrosinase activation activity was expressed as activation rate under a certain concentration. The inhibition rates (%) were calculated according to the formula as follows:

Inhibition rate (%) = [(A_Sample + LOP + TYR_ − A_Sample + LOP_)/(A_50%Me + LOP + TYR_ − A_50%Me + LOP_) − 1] × 100%

### 3.7. Partial Least Squares Analysis

The software < Chinese traditional medicine chromatographic fingerprint similarity evaluation system 2004, 1.0 A Edition > that Chinese Pharmacopoeia Commission recommended was used to correct the retention times of each peak, and the peak area was processed by equalization. Then the quantitative data were obtained. The partial least squares regression equation was established with the analysis software DPS 7.05, and the peak area was set as the independent variable (X), tyrosinase inhibition rate was taken as the dependent variable (Y). Chromatographic peaks, which were significantly correlated with inhibitory effects on tyrosinase ability, were determined, respectively.

### 3.8. Knock-Out Method

Under the optimized chromatography conditions described in section “HPLC analysis”, the water extract of AD was prepared as 1 g·mL^−1^ equivalent to raw medicinal herbs. Injection volume was 50 μL every time. The chromatogram of 312 nm was recorded. According to the peak retention time from the spectrum effect relationship analysis, the eluent solution containing the target component and the other eluent solution namely negative solution were collected, respectively. Each component was prepared and eluted in 10-fold series. The solution containing target component and negative solution were combined respectively, dissolved with 0.5 mL of 50% methanol water solution and filtered through the 0.22 μm microporous membrane, which was a sample containing target component (denoted as Sx+) and the corresponding negative sample (denoted as Sx−).

### 3.9. The High Performance Liquid Chromatography four Stage Rod-Electrostatic Field Orbit Trap High Resolution Mass Spectrometry

The compounds were detected using QExactive four stage rod-orbit trap LC-MS/MS system, containing Thermo Ultimate 3000 UHPLC system and QExactive (Thermo Fisher Scientific, Waltham, MA, USA). Separation was performed with a Waters BEH C_18_ column (2.1 mm × 50 mm, 1.7 μm; Waters, Milford, MA, USA). The mobile phase was a mixture of acetonitrile (C) and 0.1% formic acid-water (D), with an optimized linear gradient elution as follows: 0–2 min: 10–30% C; 2–10 min: 30–60% C; 10–18 min: 60–100% C; 18–25 min: 100% C; 25–26 min: 100–10% C; and 26–30 min: 10% C. The flow rate was 0.3 mL·min^−1^. The injection volume was 0.2 μL. The column temperature was set at 25 °C.

Compounds were analyzed with the full scan data in positive ion modes to provide complementary information for structural identification under the following mass spectrometry conditions: sheath gas flow rate, 35 arb; auxiliary gas flow rate, 10 arb; spray voltage, 3.5 kV; capillary temperature, 320 °C, a scan range, *m/z* 0–800 and a resolving power, 70,000. The automatic gain control (AGC) was set at 3e6 and the maximum injection time was set to 100 ms.

In addition to the full scan acquisition method, for confirmatory purpose, a targeted MS/MS analysis was also performed using the mass inclusion list and expected retention times of the target analytes, with a resolving power of 17,500. The AGC target was set to 1e5, with the maximum injection time of 50 ms. The isolation window was set at 4.0 *m/z*. Collision energy was optimized at 30 eV.

## 4. Conclusions

In this study, we investigated the components of AD that had inhibitory or activating effects on tyrosinase activity by using the spectrum-effect relationship and component knock-out method. The results showed that AD samples contained the activated and inhibitory components on tyrosinase activity simultaneously. The correlations of these components to inhibitory effects on tyrosinase activity were different, and there were either synergetic or antagonistic effects among these components. When the concentration of each sample was 1 g·mL^−1^ equal to the amount of raw medicinal herbs, oxypeucedanin hydrate, imperatorin, cnidilin, and isoimperatorin had inhibitory effects on tyrosinase activity whereas byakangelicin and bergapten had the activation effect on tyrosinase activity.

## Figures and Tables

**Table 1:** The relative retention times and characteristic peak areas of each AD sample measured by HPLC (1).

No.	S1	S2	S3	S4	S5	S6	S7	S8	S9	S10	S11
Retention Time (min)	3.874	4.519	5.909	6.845	7.675	9.514	10.339	10.985	12.728	15.923	17.119
Crude AD	201,878.5	49,559.7	75,566.7	1,014,104.0	40,144.2	72,540.4	167,879.1	20,747.8	51,836.1	23,787.2	14,466.6
50 °C-54 h	274,089.3	56,977.1	64,845.9	957,502.3	58,791.6	90,087.7	215,341.9	45,344.2	36,395.6	18,453.7	9901.8
50 °C-108 h	275,990.4	58,567.0	90,904.9	808,495.5	63,025.6	83,106.0	157,906.0	30,656.5	46,009.1	28,631.2	17,705.7
50 °C-162 h	315,305.3	78,057.7	82,778.1	1,114,690.0	80,869.4	105,522.1	215,100.8	41,739.9	51,701.6	31,061.0	26,491.5
50 °C-216 h	569,766.3	99,325.5	86,362.1	845,400.6	76,197.3	88,951.8	207,954.8	39,857.7	46,537.6	33,452.0	39,865.6
60 °C-18 h	279,296.5	46,939.6	49,067.1	903,763.3	41,834.4	41,336.5	141,072.6	22,864.6	49,040.2	16,533.9	13,715.0
60 °C-36 h	723,661.0	137,559.2	102,940.2	872,277.4	107,887.7	79,926.5	168,150.4	60,781.2	53,707.9	53,804.3	97,659.1
60 °C-54 h	345,492.1	79,299.1	39,820.8	909,679.3	72,393.7	21,835.1	167,554.0	65,991.4	57,238.7	54,571.2	35,946.3
60 °C-72 h	396,511.4	142,827.3	99,984.9	1171,416.0	100,843.1	83,110.8	311,748.7	63,046.6	76,277.5	54,851.7	108,015.9
70 °C-6 h	392,682.2	102,503.9	94,050.1	2021,756.0	83,523.6	82,903.6	276,489.5	46,788.4	48,141.3	71,767.5	30,586.5
70 °C-12 h	494,000.1	127,125.0	101,291.9	2029,405.0	127,627.5	118,232.6	304,100.3	75,638.7	136,086.9	47,603.2	75,526.9
70 °C-18 h	363,133.8	73,847.2	61,975.7	1106,638.0	30,377.1	42,254.1	211,790.0	47,271.6	46,679.7	33,697.5	35,465.3
70 °C-24 h	564,168.3	165,168.1	91,993.4	986,123.8	98,968.4	71,584.8	178,889.0	39,096.1	58,733.2	33,077.2	152,302.8
80 °C-2 h	299,940.1	66,582.1	95,923.0	1429,665.0	82,019.9	77,984.3	215,108.4	51,005.9	47,656.2	19,141.8	17,243.9
80 °C-4 h	385,776.7	64,901.7	48,255.2	1081,342.0	33,958.3	44,403.4	206,816.9	27,049.2	54,018.4	19,506.1	30,079.6
80 °C-6 h	454,612.6	105,026.1	53,240.2	962,914.4	32,132.5	37,786.3	170,128.9	27,904.2	49,049.1	35,179.2	81,775.9
80 °C-8 h	604,272.4	148,565.6	95,748.7	1205,098.0	94,460.4	68,905.1	180,280.8	41,765.0	42,885.2	30,561.8	99,850.3

**Table 2:** The relative retention times and characteristic peak areas of each AD sample measured by HPLC (2).

No.	S12	S13	S14	S15	S16	S17	S18	S19	S20	S21	S22
Retention Time (min)	18.197	18.641	19.735	20.928	22.336	22.782	24.863	26.320	27.796	29.026	29.570
Crude AD	84,836.8	14,171.4	37,971.0	407,385.4	33,890.2	150,108.6	103,350.4	1,429,522.0	20,957.4	77,836.5	10,647.0
50 °C-54 h	44,530.6	16,748.4	52,476.8	241,101.3	27,439.4	26,477.9	50,068.4	1,097,831.0	8068.8	48,917.6	8829.5
50 °C-108 h	55,860.6	19,398.1	15,753.1	122,532.9	16,906.7	34,073.7	12,089.2	1,174,544.0	2854.0	72,153.2	10,267.8
50 °C-162 h	69,561.6	14,348.0	45,258.2	197,855.1	29,180.7	32,922.1	66,647.6	951,701.6	3915.4	59,577.7	12,331.5
50 °C-216 h	43,309.0	13,456.2	32,938.5	160,425.6	31,763.4	19,361.4	286697	1,674,816.0	5508.3	52,839.8	12,526.4
60 °C-18 h	41,529.1	16,835.2	27,048.8	161,306.3	31,481.4	17,533.0	18,218.8	1,436,913.0	3830. 6	57,297.2	12,285.0
60 °C-36 h	73,923.6	23,251.7	25,035.6	125,539.7	31,413.2	24,008.3	22,679.9	1,338,823.0	5622.1	84,396.5	11,089.1
60 °C-54 h	62,818.0	23,929.9	35,879.0	175,633.9	28,180.5	17,745.6	19,747.9	1,016,496.0	4065.7	29,684.9	5563.8
60 °C-72 h	60,445.5	23,235.9	51,672.0	223,536	40,161.5	33,963.0	71,677.2	1,618,591.0	6809.8	61,019.8	13,842.3
70 °C-6 h	135,239.5	28,898.6	25,611.1	306,813.5	58,857.5	52,820.4	55,165.4	1,228,933.0	9936.0	67,869.8	15,144.1
70 °C-12 h	75,048.3	27,507.4	44,123.1	221,788.8	33,937.4	50,262.3	71,447.7	1,057,936.0	8441.7	55,721.9	5693.6
70 °C-18 h	66,896.9	29,243.2	38,535.4	204,178.3	33,451.5	21,284.0	22,426.9	1,324,433.0	4951.6	32,892.6	15,636.2
70 °C-24 h	50,777.0	18,936.5	22,319.5	146,009.3	28,044.3	18,706.5	24,005.6	1,092,613.0	4536.4	55,650.2	15,300.7
80 °C-2 h	82,418.9	21,879.2	42,242.3	273,006.3	25,581.8	72,237.3	55,817.4	942,006.6	8847.9	59,259.5	13,583.2
80 °C-4 h	56,773.6	24,274.1	36,762.0	284,134.4	28,171.6	31,174.8	19,970.9	939,136.9	9285.0	54,445.8	11,868.9
80 °C-6 h	47,258.2	22,064.1	36,628.4	134,380.3	31,220.1	17,921.7	20,012.3	1,131,498.0	3128.4	51,519.4	6042.0
80 °C-8 h	58,657.9	30,184.4	30,647.1	176,191.1	32,031.2	27,191.7	26,631.3	1,259,809.0	4381.8	45,670.7	14,184.0

**Table 3:** The relative retention times and characteristic peak areas of each AD sample measured by HPLC (3).

No.	S23	S24	S25	S26	S27	S28	S29	S30	S31	S32
Retention Time (min)	31.563	32.423	34.010	34.539	38.207	40.534	44.779	46.637	48.727	49.215
Crude AD	560,224.6	972,543.3	324,065.4	12,375.4	15,736.7	79,450.6	38,132.1	221,740.0	74,890.7	53,851.1
50 °C-54 h	592,867.9	952,622.1	359,651.1	9959.8	12,801.1	50,618.0	15,379.2	194,528.8	76,754.9	55,754.0
50 °C-108 h	523,442.3	671,940.9	206,552.5	6730.8	12,304.7	32,556.7	7312.1	125,685.2	38,401.4	35,661.4
50 °C-162 h	547,212.2	798,756.6	297,107.3	12,310.5	10,733.5	50,606.9	7524.8	147,790.4	61,352.6	49,594.6
50 °C-216 h	698,967.6	791,620.5	237,683.6	6392.9	15,180.0	41,922.0	4493.3	138,720.0	53,250.9	36,872.8
60 °C-18 h	598,011.3	817,165.9	226,284.2	4002.6	16,522.4	43,831.0	5371.3	146,085.0	53,376.8	35,968.6
60 °C-36 h	671,001.0	1,559,512.0	517,576.6	7811.5	15,443.2	49,068.4	14,457.4	252,143.1	154,725.1	76,879.2
60 °C-54 h	550,049.8	696,035.7	191,708.6	8854.8	9577.9	59,635.0	4772.7	117,058.2	49,246.5	30,484.4
60 °C-72 h	684,153.5	1,022,686.0	319,185.7	8,420.6	16,577.5	96,335.4	8999.1	185,007.3	87,662.8	51,117.9
70 °C-6 h	888,239.6	1,149,116.0	356,770.1	45,253.0	14,881.3	59,347.8	20,490.8	254,956.0	103,836.8	53,336.2
70 °C-12 h	515,345.2	1,038,435.0	324,011.2	3870.5	12,361.4	70,781.8	7478.0	143,041.9	76,715.8	45,315.0
70 °C-18 h	515,812.7	855,437.6	226,695.7	12,388.8	13,938.8	63,813.1	7266.0	142,116.2	55,039.4	33,938.2
70 °C-24 h	528,147.1	717,764.8	242,327.2	7423.3	10,147.6	52,933.8	7588.3	129,583.3	50,142.5	38,381.5
80 °C-2 h	529,400.1	1,081,962.0	331,856.6	16,461.1	16,206.7	79,560.9	8972.2	235,615.9	81,353.9	45,462.3
80 °C-4 h	481,003.8	715,983.4	211,356.3	4006.6	10,224.1	70,918.2	8020.7	118,561.2	37,647.7	31,555.4
80 °C-6 h	493,469.0	959,465.1	289,452.9	7777.3	10,934.7	55,410.2	9575.2	134,500.4	68,113.8	45,316.9
80 °C-8 h	514,575.9	714,618.2	174,512.4	8516.8	12,392.8	57,624.0	6510.2	109,653.8	54,442.2	28,237.6

**Table 4:** The relative retention times and characteristic peak areas of each AD sample measured by HPLC (4).

No.	S33	S34	S35	S36	S37	S38	S39	S40	S41	S42
Retention Time (min)	52.975	57.024	57.712	59.902	61.564	63.915	69.301	73.914	80.610	86.761
Crude AD	50,927.6	104,182.2	98,552.5	15,237.1	223,590.3	129,231.9	39,349.3	233,160.5	163,906.4	80,309.9
50 °C-54 h	61,884.9	121,212.5	77,736.0	15,603.0	173,632.8	108,427.6	28,718.7	169,335.6	171,291.8	68,429.0
50 °C-108 h	50,695.9	77,357.7	81,845.5	6325.0	196,267.5	95,585.5	29,278.7	150,975.6	107,235.6	39,845.1
50 °C-162 h	57,769.9	99,849.1	69,414.0	11,536.5	163,271.2	95,884.5	26,292.0	125,074.5	127,355.6	45,658.7
50 °C-216 h	52,017.2	86,033.1	81,737.7	6161.6	196,815.6	79,059.4	23,188.8	102,873.3	79,174.49	23,643.8
60 °C-18 h	55,377.1	76,850.4	106,775.5	13,403.5	249,757.4	120,852.6	35,528.9	147,422.0	113,002.2	32,847.6
60 °C-36 h	46,064.2	168,804.0	66,507.5	20,226.8	158,937.3	110,019.5	31,767.1	126,495.3	103,918.3	66,471.0
60 °C-54 h	66,850.1	66,097.1	96,060.6	8898.1	210,225.6	104,151.9	29,404.0	137,078.3	142,889.0	38,094.2
60 °C-72 h	64,494.1	116,323.1	104,203.6	5167.0	237,645.0	103,592.8	29,812.4	167,396.3	128,140.2	45,190.8
70 °C-6 h	94,353.5	126,491.1	76,047.4	20,075.6	157,759.3	99,033.5	25,914.1	158,990.7	212,661.6	68,108.6
70 °C-12 h	54,847.3	97,420.0	71,124.9	9211.7	149,427.0	104,972.3	28,759.3	127,258.2	121,153.1	60,246.6
70 °C-18 h	69,949.1	80,301.0	122,020.0	8565.0	265,616.2	126,703.1	37,832.2	217,166.1	166,001.8	52,442.1
70 °C-24 h	46,707.2	81,846.3	65,079.2	8844.5	152,320.3	82,649.1	22,604.9	99,800.0	82,473.0	36,173.8
80 °C-2 h	60,933.6	100,513.7	74,468.1	21,270.0	155,195.0	111,084.7	30,547.7	142,233.2	142,818.2	52,632.0
80 °C-4 h	60,916.4	67,297.8	84,519.6	10,242.4	172,952.2	97,036.15	26,573.2	145,393.5	139,343.8	41,105.5
80 °C-6 h	56,125.2	94,188.2	90,992.7	9866.5	205,797.5	125,516.2	36,502.4	160,414.3	127,849.3	51,528.5
80 °C-8 h	58,813.7	72,510.2	108,250.4	17,090.3	241,306.9	99,497.3	30,808.6	178,600.2	138,638.6	41,439.8

**Table 5:** Effect of different temperatures and heat treatment times on AD on tyrosinase activity.

Sample	Concentration of Water Extract (Equal to the Amount of Raw Medicinal Herbs)				
	1 g·mL^−1^	0.5 g·mL^−1^	0.25 g·mL^−1^	0.125 g·mL^−1^	0.0625 g·mL^−1^
Crude AD	27.07 ± 0.47	13.42 ± 0.98	9.14 ± 0.83	12.31 ± 1.16	17.29 ± 1.47
50 °C-54 h	20.84 ± 0.65 ***	12.60 ± 0.83	11.85 ± 0.41	13.53 ± 0.90	15.83 ± 0.55
50 °C-108 h	18.43 ± 0.26 ***	11.86 ± 0.68	14.94 ± 1.23	16.00 ± 0.48	4.82 ± 0.40
50 °C-162 h	15.49 ± 0.38 ***	10.09 ± 0.86	14.73 ± 0.66	15.25 ± 0.17	16.80 ± 1.52
50 °C-216 h	14.48 ± 0.46 ***	0.86 ± 0.04	−6.16 ± 0.33	4.91 ± 0.24	10.05 ± 0.22
60 °C-18 h	13.49 ± 0.46 ***	7.97 ± 0.51	4.67 ± 0.18	7.35 ± 0.53	12.44 ± 0.11
60 °C-36 h	9.43 ± 0.42 ***	−3.72 ± 0.27	−8.36 ± 0.28	−8.33 ± 0.36	−5.17 ± 0.51
60 °C-54 h	11.50 ± 0.44 ***	5.29 ± 0.48	7.03 ± 0.68	10.88 ± 0.37	15.16 ± 0.42
60 °C-72 h	7.40 ± 0.44 ***	−6.21 ± 0.16	−7.38 ± 0.17	−7.32 ± 0.33	7.50 ± 0.43
70 °C-6 h	7.12 ± 0.44 ***	−2.82 ± 0.14	−4.33 ± 0.42	−0.28 ± 0.01	4.56 ± 0.32
70 °C-12 h	4.90 ± 0.45 ***	−5.34 ± 0.30	−7.46 ± 0.28	−3.86 ± 0.29	−8.56 ± 0.32
70 °C-18 h	0.60 ± 0.06 ***	−5.48 ± 0.53	−3.53 ± 0.32	−3.24 ± 0.21	−0.12 ± 0.01
70 °C-24 h	0.55 ± 0.05 ***	−5.30 ± 0.28	3.82 ± 0.14	7.53 ± 0.60	5.70 ± 0.30
80 °C-2 h	6.73 ± 0.43 ***	−3.26 ± 0.30	−7.23 ± 0.43	−9.05 ± 0.32	−3.10 ± 0.03
80 °C-4 h	8.88 ± 0.50 ***	−4.78 ± 0.18	−1.12 ± 0.06	−0.68 ± 0.03	4.47 ± 0.28
80 °C-6 h	11.38 ± 0.44 ***	−4.96 ± 0.23	0.74 ± 0.04	1.39 ± 0.10	12.11 ± 0.43
80 °C-8 h	15.43 ± 0.47 ***	5.47 ± 0.18	4.38 ± 0.32	10.81 ± 0.76	2.67 ± 0.08

Note: compare with crude AD, * *p* < 0.05, ** *p* ≤ 0.01, *** *p* ≤ 0.001.

**Table 6:** Inhibitory effects of the water extract of AD knocked-out components and negative samples on tyrosinase in vitro.

Peak No.	Inhibitory Rate (%) on Tyrosinase Activity		
	Crude AD	Sx+	Sx−
S19	17.50 ± 0.37	−5.73 ± 0.13	25.42 ± 0.31
S21	17.50 ± 0.37	−2.72 ± 0.26	23.44 ± 0.42
S23	17.50 ± 0.37	4.28 ± 0.25	20.52 ± 1.35
S24	17.50 ± 0.37	11.32 ± 0.67	20.28 ± 0.88
S25	17.50 ± 0.37	−1.40 ± 0.30	-8.01 ± 0.41
S28	17.50 ± 0.37	5.73 ± 0.37	2.45 ± 0.19
S30	17.50 ± 0.37	−2.31 ± 0.19	29.83 ± 1.48
S31-32	17.50 ± 0.37	−1.83 ± 0.15	17.59 ± 0.68
S33	17.50 ± 0.37	−4.95 ± 0.38	19.54 ± 1.56
S34-35	17.50 ± 0.37	6.44 ± 0.18	21.26 ± 0.57
S37	17.50 ± 0.37	6.15 ± 0.24	32.37 ± 2.83
S38	17.50 ± 0.37	−13.70 ± 0.39	12.86 ± 0.55
S39	17.50 ± 0.37	−1.36 ± 0.18	24.61 ± 1.69
S40	17.50 ± 0.37	5.76 ± 0.26	34.43 ± 0.36
S41	17.50 ± 0.37	10.93 ± 1.02	−2.37 ± 0.12
S42	17.50 ± 0.37	16.71 ± 0.49	22.84 ± 0.97

**Table 7:** Settings of the time and temperature of Classic Isothermal Acceleration.

50 °C	60 °C	70 °C	80 °C
0 h	0 h	0 h	0 h
54 h	18 h	6 h	2 h
108 h	36 h	12 h	4 h
162 h	54 h	18 h	6 h
216 h	72 h	24 h	8 h

**Table 8:** The time program of gradient elution.

t/min	A/%	B/%
0	11	89
20	22	78
40	34	66
44	38	62
60	39	61
65	42	58
75	43	57
85	45	55
108	65	35

## References

[1] Chinese Pharmacopoeia Commission (2015). Chinese Pharmacopoeia.

[2] Li B., Zhang X., Wang J., Zhang L., Gao B.W., Shi S.P., Wang X.H., Li J., Tu P.F. (2014). Simultaneous characterisation of fifty coumarins from the roots of *Angelica dahurica* by off-line two-dimensional high-performance liquid chromatography coupled with electrospray ionisation tandem mass spectrometry. Phytochem. Anal..

[3] Kang J., Zhou L., Sun J., Han J., Guo D.A. (2008). Chromatographic fingerprint analysis and characterization of furocoumarins in the roots of *Angelica dahurica* by HPLC/DAD/ESI-MS^n^ technique. J. Pharm. Biomed. Anal..

[4] Liu R., Li A., Sun A. (2004). Preparative isolation and purification of coumarins from *Angelica dahurica* (Fisch. ex Hoffn) Benth, et Hook.f (Chinese traditional medicinal herb) by high-speed counter-current chromatography. J. Chromatogr. A.

[5] Xiong Y.J., Yang Y.M., Jiang S., Fang P.F., Zhao X.Y. (2010). Advances in the study of coumarins and their pharmacological actions. Chin. Tradit. Pat. Med..

[6] Duan Z.F., Chen J.W., Li X. (2008). Current research status of coumarin and their pharmacological actions in medicinal plants of umbrella family. Chin. Pharm..

[7] Yang X. (2015). The Experimental and Clinical Research of Compound Angelica Tincture in Treating Vitiligo. Master’s Thesis.

[8] Chen C.Y., Lin L.C., Yang W.F., Bordon J., Wang H.D. (2015). An updated organic classification of tyrosinase inhibitors on melanin biosynthesis. Curr. Org. Chem..

[9] Man K.H., Min H.H., Park C., Choi Y.H. (2014). Inhibitory effects of *Phyllostachys bambusoides* on melanin synthesis and tyrosinase activity in cultured human melanoma cells. J. Life Sci..

[10] Quan X.L., Zhang G.Z. (2015). Whitening effectiveness of seven white cream. Jilin J. Tradit. Chin. Med..

[11] Li C.Q., Peng L.N., Yao C., Lu C.C., Kang W.Y., Wang J.M. (2016). Effect of heating treatment on stability of two coumarins in *Angelica dahurica* and activity of tyrosinase. China J. Chin. Mater. Med..

[12] Xie J.J., Lin L., Liu S.G., Wu X.J., Ruan H.D., Guo P.X., Tong P.F., Fang B., Chen W.Y., She S.Y. (2014). Effects of imperatorin on Rab27a and tyrosinase incultured human epidermalmelanocytes. Carcinog. Teratog. Mutagen..

[13] Hu D.Q., Hou L.N., Fu R.Q., Meng D.S., Song H.Y., Xiang M.F., Xu J. (2012). Inhibition of extracts from *Angelica dahurica* on tyrosinase in vitro. China Pharm..

[14] Xie J.B. (2011). Effects of Color-Effect Herbs Medicine on Tyrosinase Activity in Cultured B16 Melanoma Cells. Ph.D. Thesis.

[15] Zhou D., Zhang J.H., Li H., Lu P.P., Wang T. (2007). Comparative study on effects of different extracts from *Angelica dahurica* on melanin. Acta. Univ. Tradit. Med. Sin. Pharmacol. Shanghai.

[16] Xu G.L., Xie M., Yang X.Y., Song Y., Yan C., Yang Y., Zhang X., Liu Z.Z., Tian Y.X., Wang Y. (2014). Spectrum-effect relationships as a systematic approach to traditional chinese medicine research: Current status and future perspectives. Molecules.

[17] Hertzberg R.P., Pope A.J. (2000). High-throughput screening: New technology for the 21st century. Curr. Opin. Chem. Biol..

[18] Zheng Q.F., Zhao Y.L., Wang J.B., Liu T.T., Zhang B., Gong M., Li J.Y., Liu H.H., Han B., Zhang Y.M. (2014). Spectrum-effect relationships between UPLC fingerprints and bioactivities of crude secondary roots of *Aconitum carmichaelii* Debeaux (Fuzi) and its three processed products on mitochondrial growth coupled with canonical correlation analysis. J. Ethnopharmacol..

[19] Cui F., Han Z.H., Liu X.H., Yang Y.F., Lan Z.H., Feng S.L. (2016). Spectrum-effect relationship of active fraction from Hedysari Radix on improving immunity. Chin. Tradit. Herb. Drugs.

[20] Xiao S. (2013). A Method for Research of Antibacterial Constituent Recognition of Traditional Chinese Medicine (*Acalypha Australis* Linn.) by Spectrum-Effect Relationship. Ph.D. Thesis.

[21] Zhao X.M., Pu S.B., Zhao Q.G., Gong M., Wang J.B., Ma Z.J., Xiao X.H., Zhao K.J. (2016). Preliminary study on effective components of *Tripterygium wilfordii forliver* toxicity based on spectrum-effect correlation analysis. China J. Chin. Mater. Med..

[22] Xu J.J. (2014). Studies in Quality Control and Evaluation Method of Menthae Haplocalycis Herba Based on the Correlation between Its Chromatographic Fingerprint and Antioxidant Activity. Master’s Thesis.

[23] Wang Q.S. (2011). Study on the Spectrum-Effect Relationship of Bupluerum Chinense and GC-MS Analysis of Supercritical-CO_2_ Fluid Extraction. Master’s Thesis.

[24] Li C.Q., Yao C., Zhu R.Y., Huang Y.X., Kang W.Y., Wang J.M. (2016). Spectrum-effect relationship in antioxidant activity of *Ligustri Lucidi* Fructus based on DPPH, ABTS and FRAP assays. China J. Chin. Mater. Med..

[25] Ye Y., Chen C.G., Lin X. (2005). Theory and application of Partial least squares regression. Strait J. Prev. Med..

[26] Shao X., Zhu K.X., Zhang Q., Zhao Y.N. (2016). Application of ingredient knock-out technology in pharmacodynamic material basis research of traditional chinese medicine. World Sci. Technol./Mod. Tradit. Chin. Med. Mater. Med..

[27] Nugroho A., Choi J.K., Park J.H., Lee K.T., Cha B.C., Park H.J. (2009). Two new flavonol glycosides from *Lamium amplexicaule* L. and their in vitro free radical scavenging and tyrosinase inhibitory activities. Planta Med..

[28] Piao X.L., Baek S.H., Park M.K., Park J.H. (2004). Tyrosinase-inhibitory furanocoumarin from *Angelica dahurica*. Biol. Pharm. Bull..

[29] Zhang W., Yin Z.H., Peng T., Kang W.Y. (2014). Effect of high-temperature heat treatment of *Psoralea corylifoliaon* the activity of tyrosinase. Chin. J. Exp. Tradit. Med. Form.

